# Influence of *Corynebacterium pseudotuberculosis* infection on level of acute phase proteins in goats

**DOI:** 10.1186/s12917-016-0675-y

**Published:** 2016-03-09

**Authors:** Z. K. H. Jeber, Z. MohdJin, F. F. Jesse, A. A. Saharee, J. Sabri, R. Yusoff, H. Wahid

**Affiliations:** Department of Veterinary Clinical Studies, Faculty of Veterinary Medicine, UPM, Serdang, Selangor Malaysia; Department of Clinical Studies, Faculty of Veterinary Medicine, UMK, Kelantan, Malaysia

**Keywords:** Caseous lymphadenitis, *Corynebacterium pseudotuberculosis*, Phospholipase D, Goat, Acute phase response, Haptoglobin, Serum amyloid A

## Abstract

**Background:**

Goat caseous lymphadenitis (CLA) is a chronic disease caused by *Corynebacterium pseudotuberculosis*. However, there is paucity of data about goat’s acute phase response during the course of CLA. This study was conducted to investigate the response of acute phase proteins, mainly haptoglobin (Hp), serum amyloid A (SAA) and the negative acute phase response, especially albumin after an experimental challenge of *C. pseudotuberculosis* and phospholipase D (PLD) in Cross bred Boer goats.

**Results:**

Serum Hp concentration in goats challenged with *C. pseudotuberculosis* (inoculated with 1x10^9^ cfu subcutaneously) showed a significant increase, 5 fold in males (0.98 ± 0.12 mg/ml) and 3 fold in females (0.66 ± 0.12 mg/ml) compared to the control (0.2 ± 0.02 mg/ml). Challenge with PLD (1 ml/20 kg body weight intravenously) also showed significant increase, 4 fold in males and females (0.89 ± 0.11 mg/ml; 0.82 ± 0.12 mg/ml) respectively compared to the control (0.2 ± 0.02 mg/ml). Albumin concentration showed a significant decrease in both treated groups compared to the control. There were no significant changes in SAA concentration between challenged and control goats.

**Conclusions:**

There was a significant response by Hp to *C. pseudotuberculosis* infection and PLD challenge. This was supported by the early acute response in which Hp was detected before CLA lesions were developed. Therefore, it concluded that *C. pseudotuberculosis* and PLD can influence the level of acute phase proteins in goats.

## Background

Caseous lymphadenitis in goats distributed throughout almost the whole world. Goat CLA is found in all continents, Asia, Australia, Africa, Europe and Americas. Farmers suffer from heavy economic losses after affected carcasses are condemned at meat inspection in abattoirs as well as due to infection or death on farms [1, 2].

Caseous lymphadenitis has a long incubation period ranging between 25 and 140 days. The disease has distinct clinical manifestations when the lesions become progressive. Most common manifestations are abscesses in the superficial lymph nodes of the body and less often internally, infecting deeply laying lymph nodes and visceral organs especially mediastinal lymph nodes and the lungs [3–6]. *C. pseudotuberculosis* has a potent exotoxin, phospholipase D which is a key virulence factor in the development of CLA [7]. Carne [8] was the first to describe PLD from *C. pseudotuberculosis*. Since then it has been detected in every isolate of *C. pseudotuberculosis* studied, including both biotypes I and II from almost all mammalian species [9]. Earlier studies [5, 10, 11] suggested that at the initial stage of CLA, *C. pseudotuberculosis* parasitises macrophages and multiplies within them [3]. Phospholipase D plays a key role in infection by enabling the organism to escape the hydrolysis process within the macrophages, exerting its effect on the inner phospholipid layer of the macrophage’s cell membrane [12].

Acute phase proteins (APPs) are found in the blood and their concentration increases or decreases in response to infection, inflammation and injury. There are two types of APPs: positive APPs when their levels increase and negative APPs if their levels decrease [13]. Positive APPs increases in response to challenge and they include haptoglobin (Hp), C-reactive protein (CRP), serum amyloid A (SAA), ceruloplasmin (CP), alph 1-acid glycoprotein (AGP) and fibrinogen; while negative APPs are albumin and transferrin. Both types are synthesized by the liver upon pro-inflammatory cytokines stimulation and released directly into blood [14].

Generally, APPs contribute to body initiating the immune system in order to limit or overcome microbial growth. Acute phase proteins are considered sensitive biomarkers, but lack specificity for different infectious agents. They can also be used in diagnosis of inflammation, monitoring treatment progress, prognosis and health status screening [15]. APPs serum concentrations change in response to major pro-inflammatory cytokines such as interleukin-6 (IL-6), tumor necrosis factor (TNF) and interleukin 1-beta (IL-1-beta) [16]. These cytokines are released by various cells with immune function, such as keratinocytes, kupffer cells, mucosal epithelia and the pituitary gland, but particularly by macrophages in response to internal or external stimuli [17]. Haptoglobin, one of the alpha-globulin constituents binds to free haemoglobin (Hb), to inhibit Hb oxidative activity [18, 19]. It has several immune-modulatory functions, mediated via binding of Hp to the CD11-CD18 receptors [20, 21]. Haptoglobin has a bacteriocidal effect and it can also inhibit mast cell proliferation, stop the maturation of epidermal Langerhans cells and suppress T cell proliferation [22–24]. Serum amyloid A is an apolipoprotein, a high density lipoprotein [25]. It has several functions such as detoxification of endotoxins, inhibition of endothelial and lymphocyte proliferation, blood platelet aggregation and it prevents T lymphocyte adherence to extracellular matrix proteins [26]. Serum amyloid A recruits the immune cells to the site of the infection and it may play a key role in inhibiting myeloperoxidase release during phagocyte migration, down-regulating the inflammatory process [27]. It is hypothesised that PLD challenge will stimulate APPs response in goat using the same pattern like from *C. pseudotuberculosis* infection. Thus, the aim of this study was to investigate and compare the influence of *C. pseudotuberculosis* infection and PLD challenge on the level of Hp and SAA in goats.

## Methods

### Ethics statement

The experiment was conducted according to the guidelines and approval of the Institutional Animal Care and Use Committee (IACUC) of Universiti Putra Malaysia, UPM (UPM/FPV/PS/3.2.1.551/AUP-R119).

### Isolation and identification of *C. pseudotuberculosis*

*C. pseudotuberculosis* was isolated from clinical cases of caseous lymphadenitis in goats. Isolates were sent to the Veterinary Laboratory Service Unit (VLSU), Department of Veterinary Pathology and Microbiology, Faculty of Veterinary Medicine, Universiti Putra Malaysia for identification and confirmation of the bacteria according to principles and methods described in the microbiological diagnostic laboratory at the Veterinary Medical Teaching Hospital, University of California, Davis, Revised Edition 2008.

### Extraction of phospholipase D

Phospholipase D, was extracted following the method described by Zaki [28]. Briefly, 2 or 3 loops of a 48 h culture of *C. pseudotuberculosis* were inoculated in a flask of freshly prepared bovine heart-liver medium. The flask was incubated anaerobically for 7 days at 37 °C in slanting position of 15° to 20°. The culture that developed a pellicle was used. Phospholipase D separation started with centrifugation of the culture medium at 8000 rpm/15 min in refrigerated centrifuge. The supernatant was collected and passed via sterile cellulose membrane filter (0.2 μm) and stored at 4C^o^ then used in the experiment. The centrifugation sediment was checked for its purity by sub-culturing it on several blood agar plates and the supernatant (PLD) was checked for sterility by incubating a bottle of 50 ml at 37C^o^ for 5 days. Then PLD was tittered for its potency using double dilution technique, washed bovine red blood cells and β-lysine from *Staphylococcus aureus*.

### Experimental inoculations

Twenty six cross bred Boer goats (13 bucks and 13 does) aged between 12 and 14 months with no history of vaccination against CLA were screened twice (3 months apart) for CLA using agar gel immunodiffusion test (AGID) prior to the experiment. The goats were divided randomly into 3 groups; the 1^st^ group consisted of 6 goats (3 males and 3 females) housed separately (to avoid mating and pregnancy hormonal changes) and inoculated with 1 ml PBS subcutaneously as control. The 2^nd^ group consisted of 10 goats (5 males and 5 females housed separately) was inoculated with *C. pseudotuberculosis* 1x10^9^ cfu subcutaneously; the 3^rd^ group also consisted of 10 goats (5 males and 5 females housed separately) injected with PLD 1 ml/20 kg body weight intravenously [29].

### Blood collection and analysis

Serial blood collections were done at 1 h, 3 h, 5 h, 8 h, 12 h then every 24 h post inoculation for 30 days during which the animals’ heath was monitored. Serum amyloid A concentration was measured using an ELISA kit (multi-species solid phase sandwich ELISA assay). Haptoglobin serum concentration was measured using an ELISA kit (multi-species colorimetric quantitative assay) with Hp control precision kit (Catalogue No: TP801-Con), all from Tridelta Development Ltd (Ireland). An ELISA reader machine from (BioRad) to read the optic density (OD) of both acute phase reactants.

### Statistical analysis

Data were analyzed using SPSS version 19.0. One way analysis of variance (ANOVA) was used with Duncan post hoc multiple comparisons. All values were reported as mean ± SE at *P* < 0.05.

## Results

### Haptoglobin

Mean serum concentrations of haptoglobin showed significant increase (*p* < 0.05) in both *C. pseudotuberculosis* and phospholipase D inoculated groups compared with the unchallenged goats. The mean concentration of Hp was also significantly higher (*p* < 0.05) in the males than the females. However, mean serum concentration of Hp in *C. pseudotuberculosis* inoculated males was higher than phospholipase D inoculated males; while it was the opposite in the females where mean serum concentration of Hp was significantly higher (*p* < 0.05) in phospholipase D inoculated females than *C. pseudotuberculosis* inoculated females (Table 1).

**Table 1:** Mean serum haptoglobin concentration (combination of all results) of the goats post inoculation with *C. pseudotuberculosis* and PLD; *n* = 26 (Mean ± SE)

Groups	Control	*C.pseudotuberculosis*	Phospholipase D
Male	0.2 ± 0.02	*0.98 ± 0.12	*0.89 ± 0.11
Female	0.2 ± 0.02	*0.66 ± 0.12	*0.82 ± 0.12

*Significant value *p* < 0.05, comparison between inoculated groups and the control

### Serum amyloid A

Mean serum concentration of amyloid A exhibits no significant changes (*p* > 0.05) in both treated groups compared with the control. Nevertheless, the females in *C. pseudotuberculosis* inoculated group showed higher mean serum concentration of SAA relatively to the males and to the unchallenged goats (Table 2).

**Table 2:** Mean serum amyloid A concentration (combination of all results) of the goats post inoculation with *C. pseudotuberculosis* and PLD; *n* = 26 (Mean ± SE)

Groups	Control	*C.pseudotuberculosis*	Phospholipase D
Male	148.33 ± 14.43	124.75 ± 13.06	156.25 ± 9.40
Female	145 ± 25.82	165 ± 10.32	156.25 ± 9.40

*Significant value *p* < 0.05, comparison between inoculated groups and the control

### Albumin

The mean concentration of serum albumin showed significant decrease (*p* < 0.05) in week 1 to 3 post inoculation with *C. pseudotuberculosis* and PLD compared to the control then returned to its normal concentration on week 4 (Table 3).

**Table 3:** Albumin means concentration (combination of all results) of the goats post inoculation with *C. pseudotuberculosis* and PLD; *n* = 26 (Mean ± SE)

Groups	Control	*C.pseudotuberculosis*	Phospholipase D
Weeks			
1	34.86 ± 1.04	*21.00 ± 5.75	*29.82 ± 1.17
2	35.56 ± 1.69	*21.44 ± 6.06	*27.78 ± 2.11
3	30.23 ± 2.33	*24.24 ± 6.26	*26.28 ± 1.98
4	31.76 ± 1.82	29.80 ± 3.16	31.88 ± 1.42

*Significant value *p* < 0.05. Comparison between inoculated groups and the control

## Discussion

The gold standards of CLA diagnosis is the detection of the infected animals and preventing them from disseminating the disease or infecting the uninfected animals. The classical way of CLA diagnosis is via bacterial culture and identification of the microorganism. However, this is possible only when the disease has become chronic and the clinical signs appeared as abscesses in the superficial lymph nodes [7, 30].

Acute phase proteins are part of the innate immune defense mechanism which responds primarily to any kind of infection and expressed as an increase in magnitude greater than 25 % [31]. The present study showed significant increase (*p* < 0.05) in mean serum haptoglobin concentration (Table 1) in both treated groups and this could be interpreted as the primary action of haptoglobin to restore the homeostasis in the body [32]. Bacterial infection triggered cytokines production especially by neutrophils and macrophages which increased the rate of APPs production in general and haptoglobin in particular [33].

In ovine caseous lymphadenitis model, haptoglobin (1.65 ± 0.21 g/L) and serum amyloid A (18.1 ± 5.2 mg/L) concentrations peaked after 7 days post-injection, a point at which the acute infection became chronic [34]. Haptoglobin concentrations were the highest between day 3 and 7 post inoculation and higher between day 3, 5 and 7 post inoculation with 2x10^5^ cfu of *C. pseudotuberculosis* VD57 wild strain and immunization with 250 μg CDM (chemically defined medium) antigen and 1.5 mg saponins respectively and compared to the control in sheep [35].

In the current study, mean serum haptoglobin concentration peaked up to 5 folds in the males and 3 folds in the females 14 days post-infection with *C. pseudotuberculosis* and up to 4 folds post-challenge with phospholipase D in both sexes (Table 1), these results were in accordance with those results reported by Abdullah et al. [31]. They observed increasing concentration of serum haptoglobin up to 7 folds in induced cases of haemorrhagic septicemia in Brangus heifers. The increased APPs concentrations may depend on the duration of the exposure to the organisms and the severity on the body tissues which in return increased the serum haptoglobin concentration in magnitude [36].

PLD inoculated goats showed almost similar pattern of response with *C. pseudotuberculosis* inoculated goats. This confirms the crucial role of PLD in the pathogenesis of CLA despite the absence of the microorganism, namely *C. pseudotuberculosis*. In fact, PLD is one of the main components of CLA commercial vaccines because of its well-known role in immune system activation and protection against *C. pseudotuberculosis* infection in sheep and goats [30, 37]. Eckersall et al. [34] reported increased serum haptoglobin concentration of up to 1.65 g/L within one week in induced infection of *C. pseudotuberculosis* in sheep. However, acute phase response was detected on day 1 post-inoculation [35]. Herein, we report that there was a significant difference in haptoglobin concentration between male and female goats. It is difficult to puzzle out such a phenomena. However, we hypothesized that males have higher blood volume, higher red blood cell count and higher haemoglobin concentration than females which may have contributed to higher Hp concentration.

Albumin is the major constituent of plasma proteins representing approximately 60 % of the entire plasma proteins, and it is considered as negative APP that shows decrease in its level upon response to infection [37], except in mastitis cases where albumin produced in the mammary gland and act as positive APP [38]. This study showed that serum albumin concentration was significantly low during week 1 to week 3 (Table 3). The cellular mechanism of positive APPs production is always associated with decrease in negative APPs particularly albumin [39]. The current study has confirmed the latter fact, with positive acute protein, particularly Hp significantly increased. Albumin has been described to decrease as a part of the metabolic response to injury or infection despite nutritional status [40].

Discordance between different APPs concentrations in illnesses and in diverse patients is common. One acute phase protein could be elevated while others may not. Such odd variations in APPs response may indicate that the acute phase reactants are individually regulated [41]. Extraordinarily, SAA showed no significant change (*P* > 0.05) in all treated groups (Table 2). Previous literatures showed increase in serum amyloid A concentration in a wide range of diseases in ruminants [14, 42, 43]. Echersall et al. [34] first described the SAA reaction in experimentally induced ovine CLA, but this study reported no elevation in serum amyloid A. This could be related to individual animal variation such as sex, breed, age and/or the chronic nature of CLA in goats. However, González et al. [44] reported moderate rise in Hp and no change in SAA in induced case of subacute ruminal acidosis in goats. These findings support our findings of no change of SAA post-inoculation with *C. pseudotuberculosis* and PLD in goats.

## Conclusions

The results directly showed that Hp has the higher response to the infection with *C. pseudotuberculosis* and PLD challenge compared to SAA. This suggests that *C. pseudotuberculosis* and PLD can influence the level of APPs in goats. However, SAA was less influenced in both treated groups which may indicates that SAA in goat might be of less value upon infectious or non-infectious conditions. Nonetheless, further investigation to evaluate SAA response may be needed.

## Abbreviations

CLA: Caseous lymphadenitis
Hp: Haptoglobin
SAA: Serum amyloid A
PLD: phospholipase D
*C. pseudotuberculosis*: *Corynebacterium pseudotuberculosis*
cfu: Colony forming unit
APPs: Acute phase proteins
CRP: C-reactive protein
CP: Ceruloplasmin
AGP: Alph 1-acid glycoprotein
IL-6: Interleukin-6
TNF: Tumor necrosis factor
IL-1-beta: Interleukin 1-beta
Hb: Haemoglobin
IACUC: Institutional Animal Care and Use Committee
VLSU: Veterinary Laboratory Service Unit
AGID: Agar gel immunodiffusion test
OD: Optic density
ELISA: Enzyme-linked immunosorbent assay
ANOVA: Analysis of variance
CDM: Chemically defined medium
